# Prospective evaluation of 64 serum autoantibodies as biomarkers for early detection of colorectal cancer in a true screening setting

**DOI:** 10.18632/oncotarget.7500

**Published:** 2016-02-19

**Authors:** Hongda Chen, Simone Werner, Julia Butt, Inka Zörnig, Phillip Knebel, Angelika Michel, Stefan B. Eichmüller, Dirk Jäger, Tim Waterboer, Michael Pawlita, Hermann Brenner

**Affiliations:** ^1^ Division of Clinical Epidemiology and Aging Research, German Cancer Research Center (DKFZ), Heidelberg, Germany; ^2^ Division of Molecular Diagnostics of Oncogenic Infections, German Cancer Research Center (DKFZ), Heidelberg, Germany; ^3^ Department of Medical Oncology, National Center for Tumor Diseases (NCT), Internal Medicine VI, University of Heidelberg, Heidelberg, Germany; ^4^ Department of General, Visceral and Transplantation Surgery, University of Heidelberg, Heidelberg, Germany; ^5^ GMP & T cell Therapy Unit, German Cancer Research Center (DKFZ), Heidelberg, Germany; ^6^ Division of Preventive Oncology, German Cancer Research Center (DKFZ) and National Center for Tumor Diseases (NCT), Heidelberg, Germany; ^7^ German Cancer Consortium (DKTK), German Cancer Research Center (DKFZ), Heidelberg, Germany

**Keywords:** autoantibody, diagnosis, colorectal cancer, tumor-associated antigens, screening setting

## Abstract

Novel blood-based screening tests are strongly desirable for early detection of colorectal cancer (CRC). We aimed to identify and evaluate autoantibodies against tumor-associated antigens as biomarkers for early detection of CRC. 380 clinically identified CRC patients and samples of participants with selected findings from a cohort of screening colonoscopy participants in 2005–2013 (N=6826) were included in this analysis. Sixty-four serum autoantibody markers were measured by multiplex bead-based serological assays. A two-step approach with selection of biomarkers in a training set, and validation of findings in a validation set, the latter exclusively including participants from the screening setting, was applied. Anti-MAGEA4 exhibited the highest sensitivity for detecting early stage CRC and advanced adenoma. Multi-marker combinations substantially increased sensitivity at the price of a moderate loss of specificity. Anti-TP53, anti-IMPDH2, anti-MDM2 and anti-MAGEA4 were consistently included in the best-performing 4-, 5-, and 6-marker combinations. This four-marker panel yielded a sensitivity of 26% (95% CI, 13–45%) for early stage CRC at a specificity of 90% (95% CI, 83–94%) in the validation set. Notably, it also detected 20% (95% CI, 13–29%) of advanced adenomas. Taken together, the identified biomarkers could contribute to the development of a useful multi-marker blood-based test for CRC early detection.

## INTRODUCTION

With approximately 1.4 million new cases and 700,000 deaths occurring in 2012, colorectal cancer (CRC) is the third most cancer and the fourth most common cause of death from cancer worldwide [1]. Stage at diagnosis is the most important prognostic factor, with 5-year relative survival ranging from >90% for patients with localized CRC to approximately 10% for patients with distant tumor spread [2, 3]. Randomized trials and observational studies have shown a large potential for reduction of CRC incidence and mortality by endoscopic or stool-based screening tests, such as sigmoidoscopy, colonoscopy, guaiac-based or immunochemical fecal occult blood tests [4–7].

An alternative approach for cancer screening might be blood-based screening tests. Due to their minimally invasive nature and straightforward implementation in routine medical examinations, blood tests might achieve high levels of adherence when applied in population-based screening [8, 9]. For instance, in a study by Adler and colleagues [9], the majority of participants who refused screening colonoscopy preferred a blood-based test (83%) over a stool-based test (15%) when both choices were offered. Autoantibodies against tumor-associated antigens (TAAs) were found to be present in cancer patients' blood. The mechanism behind the humoral immune responses toward TAAs is complex and not fully understood. Production of autoantibodies could be induced in responses to over-expression, mutations, or abnormal posttranslational modifications of proteins in cancer cells [10].

Autoantibodies as potential biomarkers for early detection of cancer have been intensively studied in previous studies [11-14]. Although the sensitivity of single autoantibodies for cancer detection seems to be low, higher sensitivities might be achieved by joint testing for multiple autoantibodies [11, 14]. Promising candidates were reported in some studies, but most of the findings were based on relatively small sample sizes, and lacked independent validation using prospectively collected samples from screening settings [11, 14]. In regards to CRC, validation of autoantibody markers in large prospective studies was rarely done, with only a few exceptions [15-17]. For instance, Pedersen and colleagues [15] evaluated autoantibodies against MUC1 and MUC4 in 97 prospectively collected CRC samples and matched healthy controls. However, the included CRC patients were recruited from an ovarian cancer screening program in UK rather than a true CRC screening population.

In this study, we evaluated the individual and joint diagnostic performance for CRC and its precursors through antibodies against a panel of 64 predefined autoantigens, and we validated the most promising marker combinations in independent samples of participants recruited in a true CRC screening setting.

## RESULTS

**Figure 1:**
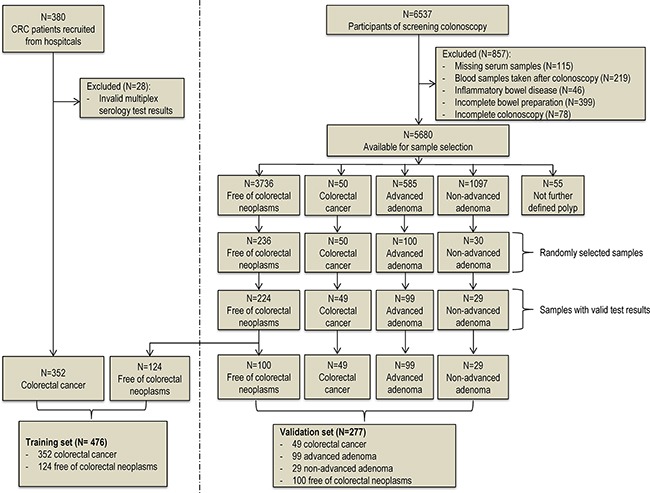
Flow diagram of sample selection procedure of the training set and the validation set.

Figure 1 provides the flow diagram showing the study population selection for the training set and the validation set. Overall, 380 clinically identified CRC cases were included in our study. After excluding 28 samples with invalid multiplex serology test results, the remaining 352 CRC patients were included as cases in the training set. The independent validation set samples were exclusively sampled from participants enrolled in the BliTz study in 2005-2013. After excluding participants without adequate blood samples, participants who do not represent a true screening setting, and participants with potentially false negative results at screening colonoscopy, 5680 participants were eligible for the sample selection, from whom 417 samples (all CRC case and random sample of advanced adenomas, non-advanced adenomas and controls free of colorectal neoplasms) were selected for our measurement. After further excluding samples with invalid laboratory results, 49 CRC cases, 99 advanced adenomas, 29 non-advanced adenomas and 224 controls free of colorectal neoplasms were included in the analysis. The average time between blood sample withdrawal and screening colonoscopy among these participants was 6.6 days. We randomly selected 124 controls from this screening setting as control group in the training set. The remaining 100 participants were used as controls in the validation set.

Table 1 presents the distribution of socio-demographic characteristics of the study population. Compared to controls, more men and older patients were included in the CRC groups of both the training set and the validation set. About half of CRC patients were diagnosed at early stages in both sets. Slightly more than half of the cancers were located in the colon (57.4% in the training set and 53.1% in the validation set). Among advanced adenomas, the proportion of polyps with high grade dysplasia (HGD), polyps with villous architecture without HGD, and large adenomas (≥10mm) with neither HGD nor villous architecture were 12%, 56%, and 32%, respectively.

**Table 1:** Characteristics of the study population

	Training set		Validation set			
Group	CRC (N, %)	Control (N, %)	CRC (N, %)	Advanced adenoma (N, %)	Non−advanced adenoma (N, %)	Control (N, %)
**Age (years)**						
**<60**	73(20.8)	50 (40.3)	9 (18.4)	30 (30.3)	10 (34.5)	43 (43.0)
**60−64**	49 (14.0)	30 (24.2)	14 (28.6)	27 (27.3)	7 (24.1)	22 (22.0)
**65−69**	57 (16.2)	22 (17.7)	11(22.4)	16 (16.2)	6 (20.7)	20 (20.0)
**≥70**	172 (49.0)	22 (17.7)	15 (30.6)	26 (26.3)	6 (20.7)	15 (15.0)
**Mean ± SD**	68.3 ± 11.6	62.2 ± 7.4	66.3 ± 6.7	64.5 ± 7.7	63.6 ± 6.5	62.0 ± 6.1
**Sex**						
**Male**	202 (57.4)	56 (45.2)	34 (69.4)	50 (50.5)	17 (58.6)	45 (45.0)
**Female**	150 (42.6)	68 (54.8)	15 (30.6)	49 (49.5)	12 (41.4)	55 (55.0)
**UICC TNM tumor stage**						
**Tis (0)**	−	−	4 (8.2)	−	−	−
**I**	96 (27.3)	−	18 (36.7)	−	−	−
**II**	102 (29.0)	−	5 (10.2)	−	−	−
**III**	105 (29.8)	−	19 (38.8)	−	−	−
**IV**	49 (13.9)	−	3 (6.1)	−	−	−
**CRC location**							
**Colon**	202 (57.4)	−	26 (53.1)	−	−	−
**Rectum**	150 (42.6)	−	22 (44.9)	−	−	−
**Unknown**	−	−	1 (2.0)	−	−	−
**Advanced adenoma subclass**						
**HGD**	−	−	−	12 (12.1)	−	−
**Villous**	−	−	−	55 (55.6)	−	−
**Large polyp**	−	−	−	32 (32.3)	−	−

Abbreviations: CRC, colorectal cancer; HGD, high grade dysplasia; SD, standard deviation; TNM, tumor-node-metastasis; UICC, Union for International Cancer Control.

**Figure 2:**
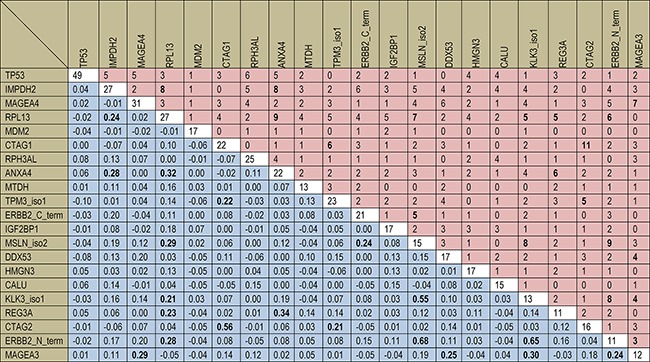
Individual and joint seropositivity of 18 autoantibody markers in the 352 CRC cases of the training set. The diagonal (white cells) and the upper right triangle (red cells) show the absolute numbers of seropositivity for single autoantibody markers and of joint seropositivity for pairwise marker combinations, respectively. The lower left triangle (blue cells) shows Cohen's Kappa coefficients for pairwise marker combinations. Bold figures indicate Cohens's Kappa coefficients higher than 0.20 and corresponding absolute numbers of joint seropositivity.

A head-to-head comparison of the diagnostic performance of all 64 autoantibody markers for detecting colorectal neoplasms is shown in Supplementary Table 2 and the diagnostic performance of the 21 best performing autoantibodies identified in the training set is presented in Table 2. Using cutoffs yielding 98% specificity in the training set generally resulted in rather low levels of sensitivity in both the training set and the validation set. The majority of markers were found to have lower sensitivities for CRC in the validation set compared to the training set. Overall, there were 21 markers showing sensitivities for early stage CRC ≥4% at 98% specificity in the training set. However 7 of them did not detect any early stage CRC in the validation set (see Table 2). We further examined the joint seropositivity of the top 21 markers among cases of the training set (see Figure 2). There was only very limited co-occurrence beyond chance of the single autoantibody markers. Although the majority of Kappa coefficients were positive (145 out of 210, 69%), 189 out of 210 Kappa coefficients (90%) for pairwise joint occurrence were close to zero with values between −0.10 and +0.20.

**Table 2:** Diagnostic performance of the top 21 single autoantibody markers for detecting colorectal neoplasms

Antigen	cutoff (MFI)	Training set				Validation set					
		SE for CRC^†^	SE for stage I/II CRC^†^	SE for stage III/IV CRC^†^	SPE^†^	SE for CRC^†^	SE for stage 0/I/II CRC^†^	SE for stage III/IV CRC^†^	SE for AA^†^	SE for NA^†^	SPE^†^
**TP53**	601	14 [11–18]	13 [9–19]	15 [10–21]	98 [93–99]	8 [3–19]	7 [2–23]	9 [3–28]	1 [0–6]	0 [0–12]	100 [96–100]
**IMPDH2**	47	8 [5–11]	10 [7–15]	5 [2–9]	98 [93–99]	4 [1–14]	4 [0–18]	5 [0–22]	6 [3–13]	7 [2–22]	95 [89–98]
**MAGEA4**	168	9 [6–12]	9 [6–14]	8 [5–14]	98 [93–99]	6 [2–17]	11 [4–28]	0 [0–15]	12 [7–20]	0 [0–12]	96 [90–98]
**RPL13**	170	8 [5–11]	9 [5–13]	6 [4–12]	98 [93–99]	4 [1–14]	0 [0–12]	9 [3–28]	2 [1–7]	10 [4–26]	97 [92–99]
**MDM2**	400	5 [3–8]	8 [5–12]	1 [0–5]	98 [93–99]	2 [0–11]	4 [0–18]	0 [0–15]	1 [0–6]	7 [2–22]	98 [93–99]
**CTAG1**	91	6 [4–9]	7 [4–11]	6 [3–11]	98 [93–99]	2 [0–11]	0 [0–12]	5 [0–22]	6 [3–13]	3 [0–17]	95 [89–98]
**RPH3AL**	87	7 [5–10]	7 [4–11]	8 [5–13]	98 [93–99]	0 [0–7]	0 [0–12]	0 [0–15]	6 [3–13]	3 [0–17]	91 [84–95]
**ANXA4**	50	6 [4–9]	6 [4–10]	6 [4–12]	98 [93–99]	4 [1–14]	4 [0–18]	5 [0–22]	2 [1–7]	7 [2–22]	99 [95–100]
**MTDH**	440	4 [2–6]	6 [4–10]	1 [0–4]	98 [93–99]	4 [1–14]	4 [0–18]	5 [0–22]	2 [1–7]	3 [0–17]	98 [93–99]
**TPM3_iso1**	424	7 [4–10]	6 [4–10]	7 [4–12]	98 [93–99]	8 [3–19]	0 [0–12]	18 [7–39]	4 [2–10]	7 [2–22]	96 [90–98]
**ERBB2_C_term**	90	6 [4–9]	6 [3–10]	6 [4–12]	98 [93–99]	6 [2–17]	7 [2–23]	5 [0–22]	8 [4–15]	3 [0–17]	92 [85–96]
**IGF2BP1**	309	5 [3–8]	6 [3–10]	4 [2–8]	98 [93–99]	2 [0–11]	4 [0–18]	0 [0–15]	2 [1–7]	3 [0–17]	99 [95–100]
**MIA**	52	4 [3–7]	5 [3–9]	3 [1–7]	98 [94–100]	2 [0–11]	4 [0–18]	0 [0–15]	1 [0–6]	3 [0–17]	98 [93–99]
**DDX53**	460	5 [3–8]	5 [2–8]	5 [3–10]	98 [93–99]	0 [0–7]	0 [0–12]	0 [0–15]	2 [1–7]	3 [0–17]	99 [95–100]
**HMGN3**	706	5 [3–8]	5 [2–8]	5 [3–10]	98 [93–99]	8 [3–19]	11 [4–28]	5 [0–22]	9 [5–16]	10 [4–26]	94 [88–97]
**CALU**	578	4 [3–7]	4 [2–8]	5 [2–9]	98 [93–99]	6 [2–17]	0 [0–12]	14 [5–33]	4 [2–10]	3 [0–17]	92 [85–96]
**KLK3_iso1**	41	4 [2–6]	4 [2–8]	3 [1–7]	98 [93–99]	2 [0–11]	4 [0–18]	0 [0–15]	1 [0–6]	3 [0–17]	98 [93–99]
**REG3A**	62	3 [2–6]	4 [2–8]	2 [1–6]	98 [93–99]	0 [0–7]	0 [0–12]	0 [0–15]	1 [0–6]	3 [0–17]	99 [95–100]
**CTAG2**	135	5 [3–7]	4 [2–7]	6 [3–11]	98 [93–99]	4 [1–14]	7 [2–23]	0 [0–15]	3 [1–9]	0 [0–12]	93 [86–97]
**ERBB2_N_term**	81	3 [2–6]	4 [2–7]	3 [1–6]	98 [93–99]	4 [1–14]	4 [0–18]	5 [0–22]	3 [1–9]	3 [0–17]	97 [92–99]
**MAGEA3**	220	3 [2–6]	4 [2–7]	3 [1–7]	98 [93–99]	4 [1–14]	7 [2–23]	0 [0–15]	6 [3–13]	0 [0–12]	95 [89–98]

† In % with 95% confidence interval.

Abbreviations: AA, advanced adenoma; NA, non−advanced adenoma, CRC, colorectal cancer; MFI, median fluorescence intensity; SE, sensitivity; SPE, specificity.

Overall, there were only 9 autoantibody markers whose sensitivities for detecting early stage CRC were higher than 5%, ranging from 7% to 11% in the validation set (see Supplementary Table 2). Anti-MAGEA4 was found to have best diagnostic potential for detecting early stage CRC and advanced adenomas, with sensitivities of 11% (95% CI, 4−28%) and 12% (95% CI, 7−20%), respectively, at a specificity of 96%. Anti-TP53 detected 8% of CRC (7% at early stage), but only 1% of advanced adenomas at 100% specificity.

A systematic search for the best n-marker combination was conducted among all the 64 autoantibody markers with the purpose to enhance the diagnostic performance. Results for the best performing 2-, 3-, 4-, 5- and 6-marker combinations are shown in Table 3. Including six or more markers in the combination resulted in specificities much lower than 90% for the best performing marker combinations even in the training set, the analysis was therefore limited to combinations of no more than 6 markers. As expected, sensitivities increased and specificities decreased with increasing numbers of markers included the multi-marker combination. For example, sensitivity for any CRC increased from 22% for the best performing 2-marker panel to 40% for the best performing 6-marker panel in the training set, whereas specificity decreased from 95% to 88%. Interestingly, sensitivity was consistently higher for early stage than for advanced stage CRC. As expected, sensitivities were slightly lower in the validation set, but a sensitivity of 30% (95% CI, 16−48%) for early stage CRC along with a specificity of 85% (95% CI, 77−91%) was still observed for the best performing 6-marker combination. Additionally, it also detected 25% (95% CI, 18−35%) of advanced adenomas, the most important precursors of CRC.

**Table 3:** Diagnostic performance of best performing two-, three-, four-, five-, and six-marker combinations derived from training set for detecting colorectal neoplasms

Marker combination	Training set				Validation set					
	SE for CRC^†^	SE for stage I/II CRC^†^	SE for stage III/IV CRC^†^	SPE^†^	SE for CRC^†^	SE for stage 0/I/II CRC^†^	SE for stage III/IV CRC^†^	SE for AA^†^	SE for NA^†^	SPE^†^
***Best performing two-marker combination***										
**TP53+IMPDH2**	22 (18-26)	23 (17-29)	20 (15-28)	95 (90-98)	10 (4-22)	11 (4-28)	9 (3-28)	7 (3-14)	7 (2-22)	95 (89-98)
***Best performing three-marker combination***										
**TP53+IMPDH2+MDM2**	26 (22-31)	30 (24-37)	21 (15-29)	93 (87-96)	12 (6-32)	15 (6-32)	9 (3-28)	8 (4-15)	10 (4-26)	94 (88-97)
***Best performing four-marker combination***										
**TP53+IMPDH2+MDM2+MAGEA4**	33 (28-38)	37 (30-44)	28 (21-36)	90 (84-94)	18 (10-31)	26 (13-45)	9 (3-28)	20 (13-29)	10 (4-26)	90 (83-94)
***Best performing five-marker combination***										
**TP53+IMPDH2+MDM2+MAGEA4+CTAG1**	38 (33-43)	43 (36-50)	32 (25-40)	89 (82-93)	20 (11-34)	26 (13-45)	14 (5-33)	24 (17-34)	14 (5-31)	87 (79-92)
***Best performing six-marker combination***										
**TP53+IMPDH2+MDM2+MAGEA4+CTAG1+MTDH**	40 (34-45)	46 (39-53)	32 (25-40)	88 (81-93)	24 (15-38)	30 (16-48)	18 (7-39)	25 (18-35)	17 (8-35)	85 (77-91)

† In % with 95% confidence interval.

Abbreviations: AA, advanced adenoma; CI, confidence interval; CRC, colorectal cancer; NA, non-advanced adenoma; SE, sensitivity; SPE, specificity.

**Figure 3:**
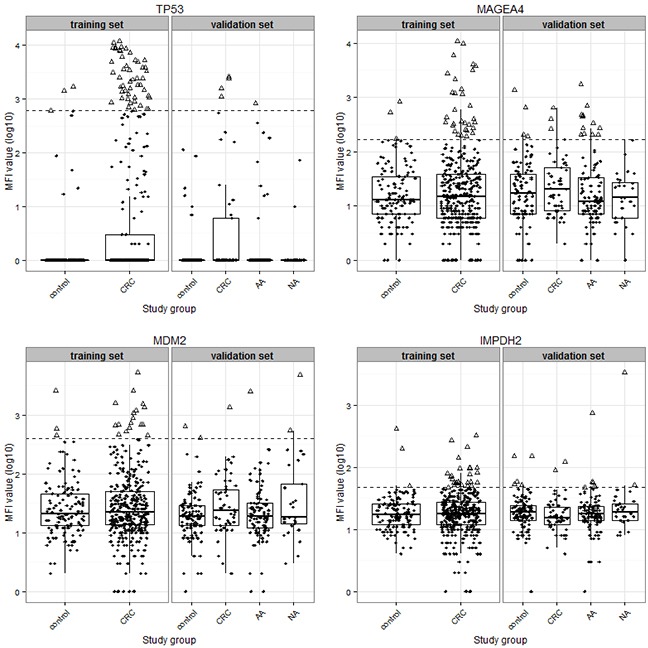
Scatter plot and box plot showing the distribution of median fluorescence intensity (MFI) values (in log10 transformation) of four autoantibody biomarkers (anti-TP53, anti-MAGEA4, anti-MDM2 and anti-IMPDH2) between different study groups. The bottom and top of the box indicate the first (Q1) and third (Q3) quartiles, and the middle line in the box is the median; the upper-limit equals Q3 plus 1.5 times interquartile range (IQR), and the lower-limit equals Q1 minus 1.5 times IQR. The horizontal dashed lines represent the cutoffs of respective biomarkers. Triangles represent samples with MFI values above the respective cutoffs, and solid dots represent samples with MFI values below the respective cutoffs. Abbreviations: AA, advanced adenoma; CRC, colorectal cancer; NA, non-advanced adenoma.

We further evaluated additional seven 6-marker combinations, whose Youden indices were only slightly lower than the best-performing 6-marker combination in the training set. Details of the diagnostic performance of these combinations are shown in Table 4. Overall, the sensitivities of these 6-marker combinations for detecting CRC ranged from 20% to 29% at specificities ranging from 84% to 86%. The sensitivities for detecting early stage CRC were higher than for detecting advanced stage CRC, and ranged from 26% to 30%. Notably, these panels also exhibited good diagnostic potential for detecting advanced adenomas, with sensitivities ranging from 21% to 27%. Interestingly, four autoantibody markers, anti-TP53, anti-IMPDH2, anti-MDM2 and anti-MAGEA4, were consistently included in all of these seven 6-marker combinations. These are also the makers that were included in the best 4-marker combination which yielded almost the same sensitivity for early stage and advanced adenomas (26% and 20%, respectively), albeit at higher specificity (90%). A graphic presentation of the MFI value distributions of these four markers is shown in Figure 3.

**Table 4:** Diagnostic performance of the top 8 best 6-marker combinations derived from training set for detecting colorectal neoplasms

Marker combination	Training set				Validation set					
	SE for CRC^†^	SE for stage I/II CRC^†^	SE for stage III/IV CRC^†^	SPE^†^	SE for CRC^†^	SE for stage 0/I/II CRC†	SE for stage III/IV CRC^†^	SE for AA^†^	SE for NA^†^	SPE^†^
**Panel 1**	40 (34-45)	46 (39-53)	32 (25-40)	88 (81-93)	24 (15-38)	30 (16-48)	18 (7-39)	25 (18-35)	17 (8-35)	85 (77-91)
**Panel 2**	42 (37-48)	48 (41-55)	35 (28-43)	86 (79-91)	27 (16-40)	26 (13-45)	27 (13-48)	27 (19-37)	21 (10-38)	84 (76-90)
**Panel 3**	39 (34-44)	44 (37-51)	32 (25-40)	89 (82-93)	24 (15-38)	30 (16-48)	18 (7-39)	21 (14-30)	21 (10-38)	86 (78-91)
**Panel 4**	39 (34-45)	44 (37-51)	33 (26-41)	89 (82-93)	27 (16-40)	26 (13-45)	27 (13-48)	23 (16-32)	17 (8-35)	85 (77-91)
**Panel 5**	39 (34-45)	45 (38-52)	32 (25-40)	88 (81-93)	22 (13-36)	30 (16-48)	14 (5-33)	24 (17-34)	14 (5-31)	85 (77-91)
**Panel 6**	41 (36-46)	46 (39-53)	34 (27-42)	87 (80-92)	20 (11-34)	26 (13-45)	14 (5-33)	26 (19-36)	14 (5-31)	86 (78-91)
**Panel 7**	41 (36-47)	46 (39-53)	35 (28-43)	87 (80-92)	24 (15-38)	26 (13-45)	23 (10-43)	25 (18-35)	21 (10-38)	85 (77-91)
**Panel 8**	42 (37-47)	47 (40-54)	36 (28-44)	86 (79-91)	29 (18-42)	26 (13-45)	32 (16-53)	24 (17-34)	24 (12-42)	85 (77-91)

† In % with 95% confidence interval.

Abbreviations: AA, advanced adenoma; CI, confidence interval; CRC, colorectal cancer; NA, non-advanced adenoma; SE, sensitivity; SPE, specificity.

Panel 1: TP53 + IMPDH2 + MDM2 + MAGEA4 + CTAG1+ MTDH

Panel 2: TP53 + IMPDH2 + MDM2 + MAGEA4 + CTAG1 + TPM3_iso1

Panel 3: TP53 + IMPDH2 + MDM2 + MAGEA4 + RPL13 + SAG

Panel 4: TP53 + IMPDH2 + MDM2 + MAGEA4 + TPM3_iso1 + CTAG1

Panel 5: TP53 + IMPDH2 + MDM2 + MAGEA4 + CTAG1 + SAG

Panel 6: TP53 + IMPDH2 + MDM2 + MAGEA4 + CTAG1 + IGF2BP1

Panel 7: TP53 + IMPDH2 + MDM2 + MAGEA4 + CTAG1 + RPL13

Panel 8: TP53 + IMPDH2 + MDM2 + MAGEA4 + TPM3_iso1 + RPL13

## DISCUSSION

In this study, we first identified autoantibody markers and their combinations for early detection of CRC and its precursors in a large set of samples, which were then independently validated in prospectively collected samples from a true screening population. Overall, most of the tested autoantibodies showed relatively low sensitivity at cutoff levels yielding high specificity. Among all single markers, anti-MAGEA4 showed the highest sensitivity for detecting early stage CRC (11%, 95% CI, 4−28%) and advanced adenomas (12%, 95% CI, 7−20%) at a specificity of 96% (95% CI, 90−98%). We also explored the potential of multi-marker combinations to enhance diagnostic performance. Sensitivity increased and specificity decreased with increasing numbers of markers included in multi-marker combinations. All marker-combinations had higher sensitivities for early stage than for advanced stage CRC even in the validation set. Four markers, including anti-TP53, anti-MAGEA4, anti-MDM2 and anti-IMPDH2, were consistently included in best performing 4-, 5-, and 6-marker combinations. A combination of these four markers could detect 26% of early stage CRC (95% CI, 13−45%) and 20% of advanced adenomas (95% CI, 13−29%) at a specificity of 90% (95% CI, 83−94%).

Many single autoantibody markers tested in our study were also evaluated in previous studies, mostly using retrospective case-control designs. Apparently substantially better discriminative ability between CRC and healthy controls were reported for some markers. For instance, Kanojia and colleagues [18] measured blood anti-SPAG9 from 54 clinically diagnosed CRC patients and 50 healthy donors using ELISA and Western Blot. They reported a sensitivity of 70% at 100% specificity. However, in our analyses, anti-SPAG9 only detected 2% of the CRC cases at 98% specificity in the training set, and 4% of the CRC cases at 97% specificity in the validation set. Similar apparent discrepancies with previous reports were observed for other markers, such as anti-RPH3AL [19], anti-SEC61B [20], anti-ANXA4 [21].

The apparent discrepancies could be explained by different design issues and different study settings adopted in different studies. Most previous studies recruited participants in clinical settings, i.e., using clinically detected cases and convenience controls or healthy donors who often substantially differed from cases with respect to major characteristics that may be related to the autoantibody levels, such as age and sex [18-21]. Under such circumstances, a number of factors, such as tumor stage [22] and size, or differences in sociodemographic characteristic between cases and controls [23] may affect the validity of study findings. Moreover, most previous studies did not take efforts to adjust for the potential overestimation of diagnostic performance indicators, either through internal validation (bootstrap or cross-validation) or through external validation. In our analyses, a two-step approach was taken including selecting biomarkers in a training set and subsequently validating findings in an independent validation set. Of note, the validation was performed in a true screening setting, i.e., in the ideal target population for CRC screening, with colonoscopic verification of presence or absence of colorectal neoplasms (including adenomas) among all participants. By adopting such a study design and analysis strategy, the pitfalls often encountered in retrospective study designs could be avoided [24, 25]. In addition, we applied multiplex Luminex-based serological assays to measure all 64 autoantibody markers. Previous research results indicated that multiplex serology exhibited increased detection of weak antibody responses compared to ELISA [26], thus possibly providing more accurate results regarding serum autoantibody responses against TAAs.

Definition of cutoff directly affects the sensitivity and specificity of respective markers. In our analysis, the cutoffs of 64 autoantibody markers were set at relatively high levels to ensure very high specificities of each single marker (≥ 98% in the training set). This may explain the somewhat lower sensitivity for the single markers compared to previous reports, but it ensured maintenance of reasonably high specificity of multi-marker combinations. For instance, anti-TP53 is a widely evaluated antibody marker for cancer detection. In the current study, ROC (receiver operating characteristic) analysis showed that the AUC (area under the curve) of anti-TP53 for detecting CRC was 0.60 in the validation set (data not shown in the results). This result was quite similar to results from other studies evaluating anti-TP53 for early detection of CRC in large prospective studies [16, 17].

Among tested single autoantibody markers, anti-MAGEA4 was found to have good diagnostic potential for detecting early stage CRC (sensitivity=11%; 95% CI, 4−28%) and advanced adenomas (sensitivity=12%; 95% CI, 7−20%) in our analysis. MAGEA4 is a member of cancer/testis (CT) antigens, which is expressed in malignant cells and in immune-privileged germ-line cells [27]. It has a putative role of mediating anti-apoptotic functions by interacting with TP53 [28]. Over-expression of MAGEA4 was previously reported in other malignancies, such as lung cancer [29] and ovarian cancer [30]. To our knowledge, our study is the first to indicate that humoral responses to MAGEA4 could be observed in the early stage of CRC and its precursors. Although its diagnostic performance as a single biomarker is quite limited, it seems to have potential to be included in a multi-marker panel in the future.

As genetic or epigenetic alterations leading to aberrantly expression of antigens, such as TP53 [31] and CTAG1 (also known as NY-ESO-1) [32], may occur in various types of cancer, humoral responses to autoantigens evaluated in our study may also be observed in patients with other malignancies rather than in CRC only. To what extent these markers could detect other malignancies should be evaluated in future studies.

The sensitivities for detecting CRC of the included autoantibodies are clearly inferior to detection of CRC by currently recommended tests for early detection of CRC, i.e., faecal immunochemical tests (FITs) for hemoglobin [33, 34], and possibly even inferior to guaiac-based faecal occult blood tests (gFOBTs) [35, 36], probably still the most widely used non-invasive tests globally. Notably, sensitivities for detecting advanced adenomas of our marker combinations (≥20%) were similar to sensitivities reported for FITs [33, 34], and clearly superior to sensitivities reported for gFOBTs^35,36^ and any of other suggested blood tests that were validated in screening settings. For instance, a recent study by Church and colleagues [37] tested methylated Septin9 in 7941 asymptomatic individuals attending screening colonoscopy, and reported a sensitivity for detecting advanced adenoma of 4.2% (6/130) and 15.7% (24/184) for women and men, respectively, at a specificity of 91.5%.In our study, we deliberately selected antibody markers against TAAs based on their ability to detect early stage CRC rather than any stage CRC. This most likely explains the apparently unusual finding of better diagnostic performance for early stage CRC than for late stage CRC even in the validation set, whereas other biomarker studies typically showed opposite patterns. Another possible explanation might be immune destruction, an emerging hallmark of cancer [38], which occurs in advanced stage of CRC inducing the loss of antibody production. In a screening setting, the vast majority of prevalent preclinical and previously undetected CRC are at an early stage [22, 39]. Early detection and removal of these cancers, along with early detection and removal of the precursors, advanced adenomas, is likely to bear the largest potential for prevention of CRC deaths [5, 40].

It should be stated that the multi-marker panels identified in this study cannot be regarded as a final solution for CRC screening due to their overall limited sensitivity. Nevertheless, given their potential for detecting early stage CRC and especially its most important precursor – advanced adenomas (positive predictive values ranging from 57% to 67%), at high specificities, combining these panels with other promising biomarkers or tests might contribute to the development of clinically useful multi-marker panel in the future. In particular, further research should also explore the possibility of enhanced detection of advanced adenomas by combining FIT with a panel of autoantibody markers. However, additional issues such as compliance to specimen sampling (blood and stool), complexity of conducting multiple tests and cost, need to be taken into consideration for such potential test combinations.

Specific strengths and limitations deserve careful consideration when interpreting our study. Strengths include that we adopted a two-step approach to minimize overestimation of indicators of diagnostic performance. The validation of biomarkers was conducted in a large subset sample of participants of screening colonoscopy, which represent the target population for CRC screening in which the diagnostic performance of biomarkers should be evaluated. Furthermore, we applied the state-of-art technique to measure a large number of autoantibodies simultaneously, enabling a head-to-head comparison of the diagnostic performance of these biomarkers possible. Limitations of our study include the relatively small number of CRC cases in the validation samples, despite the very large screening population recruited, which reflects the very low prevalence of CRC in a true screening population. Moreover, the target antigens evaluated in our measurement were mostly native full-length proteins. Other types of TAAs, such mutated neo-antigens or proteins with abnormal posttranslational modifications were not tested in our study. In addition, the cutoffs of respective autoantibody markers were defined based on MFI values of controls with no significant findings at screening colonoscopy in the training set. Further determination of optimal cutoffs of respective autoantibody markers needs to be validated in larger screening populations.In summary, autoantibodies against TAAs carry potential for early detection of CRC. The multi-marker combinations developed in our study exhibited promising diagnostic performance for detecting a relevant proportion of early stage CRC and its relevant precursors. The combination of the most promising biomarkers identified in our study with additional promising biomarkers or tests might contribute to the development of a useful multi-marker blood-based test for early detection of CRC and its precursors in the future.

## MATERIALS AND METHODS

### 1 Study design and study population

Our analysis is based on the ongoing BliTz study (Begleitende Evaluierung innovativer Testverfahren zur Darmkrebsfrüherkennung), which has been described in detail elsewhere [34, 41]. Briefly, participants of screening colonoscopy are recruited in gastroenterology practices in Southern Germany since November 2005. Patients are recruited at a preparatory visit and invited to donate blood and stool samples for the evaluation of novel CRC screening tests (typically one week) before screening colonoscopy. Colonoscopy records are collected from all participants. In Germany, screening colonoscopies have been offered for CRC screening since October 2002. Only experienced endoscopists are licenced for performing screening colonoscopies. Rigorous standards, including central review of full examination documentation, have been adopted to ensure high quality of screening colonoscopy [39, 42, 43]. The high quality of screening colonoscopy in Germany is reflected in high adenoma detection rates which have steadily increased since the introduction of the screening program [44]. Histopathological examination of polyps is decentrally conducted by certified pathology labs.

For this analysis, the following exclusion criteria were applied to exclude participants without adequate blood samples, participants not representing a true screening setting, and participants with potentially inadequate colonoscopy results: blood samples taken after screening colonoscopy, history of CRC or inflammatory bowel disease, incomplete colonoscopy or insufficient bowel preparation (latter only for controls). From the remaining participants of the BliTz study recruited in 2005-2013 (N=5680), all 50 newly detected CRC cases, as well as random samples of participants with advanced colorectal adenomas (AA, N=100), non-advanced colorectal adenomas (NA, N=30) and with no colorectal neoplasms (controls, N=236) were included in the measurement. Because in a true screening population like ours CRC patients are expected to be on average slightly older and to include a somewhat larger proportion of men, age and sex were not matched between different disease groups to avoid biased estimates of specificity [23].

Given the limited number of CRC cases even in a screening study as large as the BliTz study, we also included 380 CRC cases recruited after diagnosis but before treatment at four hospitals in Southern Germany for the discovery phase of this study. All studies were approved by the ethics committees of the medical faculty of the University of Heidelberg and of the respective state medical boards. Written informed consent was obtained from each participant.

### 2 Classification of colorectal cancer and advanced adenoma

Based on the colonoscopy reports or hospital records of all participants, invasive CRC was classified into stages Tis (carcinoma in situ) and I–IV according to the UICC (Union for International Cancer Control) TNM (tumor-node-metastasis) stage classification (7^th^ version). In addition, advanced adenomas were subclassified into high grade dysplasia (HGD), villous architecture without HGD, and large adenomas (≥10 mm) with neither HGD nor villous architecture. Relevant information was extracted by two research assistants independently who were blind to the blood test results.

### 3 Laboratory procedures

#### 3.1 Sample preparation

Blood samples were collected in Sarstedt S-Monovette (Product No. 02.1388) tubes and Vacutainer SST II (Product No. 367953) tubes before bowel preparation for colonoscopy (BliTz study), or prior to large bowel surgery or neoadjuvant therapy (380 CRC cases from the clinical setting). After completion of blood clotting, blood samples were immediately centrifuged at 2000−2500g for 10 minutes and stored at −80°C until analyses. Details on the standard operating procedures have also been described previously [45].

#### 3.2 Multiplex serology

We selected 64 candidate TAAs encoded by 59 genes based on previous autoantibody measurements [46] and two recent systematic reviews [11, 14]. Details on the 64 candidate TAAs are provided in Supplementary Table 1. Autoantibodies to the selected TAAs were measured by multiplex serology, a fluorescent bead-based GST capture immunosorbent assay, as described previously 46, 47]. In short, TAAs were bacterially expressed as GST-X-tag fusion proteins [48], loaded and affinity-purified on glutathione-casein-coupled spectrally distinct fluorescence-labeled polystyrene beads (SeroMap, Luminex Corp., Austin, Tx, USA). A mix of differently loaded bead sets provides an antigen suspension array that is presented to sera. A Luminex analyzer (Luminex Corp., Austin, Tx, USA) distinguishes the bead set by its internal bead-color and quantifies the amount of serum-antibody detected by a secondary goat antihuman IgA, IgM, IgG antibody (Dianova, Hamburg, Germany) and the reporter conjugate streptavidin-R-phycoerythrin. The antibody reactivity is given as median fluorescence intensity (MFI) of at least 100 beads per set measured. Final antigen-specific MFI values were generated by subtraction of GST-tag and individual bead background values.

Sera were sent on dry ice to the Division of Molecular Diagnostics of Oncogenic Infections, DKFZ, Heidelberg and analyzed in a 1:1000 dilution by laboratory staff that was blinded for the case-control status.

Antigen-loading of the beads was controlled via detection of the C-terminal tag. Identity of the antigen loaded on the beads was verified by identifying the encoding plasmids via PCR and sequencing. Serum samples were denominated as invalid due to either insufficient amount of serum or too high GST-background values (>300 MFI). Variation between different assay-plates was controlled by pipetting three control sera on each plate as replicates. For overall 19 replicates, coefficients of variation (CV) were calculated for every antigen with a mean reactivity above 30 MFI. The median (range) CVs for the three samples were 16% (10-24%), 14% (11-21%), and 18% (11-25%), respectively.

### 4 Statistical analyses

We used a two-step approach with selection of markers and marker combinations in a training set, and validation of the results in an independent validation set. The validation set was defined in such a way that it represents a true screening setting, i.e., only participants from the BliTz study were included. CRC cases recruited in the clinical setting and randomly selected controls from the BliTz study were used for the training set.

Characteristics of the study population were first described and compared between the training set and the validation set. The strategy for selection of autoantibodies and their cutoff levels for predicting presence of colorectal neoplasm were based on the particular character of single autoantibody markers, which typically have rather low sensitivity at very high specificity [11, 14]. Cutoff levels yielding 98% specificity in the training set were determined (cutoffs lower than 30 MFI were replaced by a technical minimum cutoff of 30 MFI to reduce the influence of background noise of the multiplex serology measurement). After applying the cutoffs to respective markers, dichotomous variables indicating the predicted outcome (positive or negative) were generated. Sensitivities of all the 64 autoantibody markers for detecting CRC were calculated. Additionally, multi-marker combinations were also explored to enhance the diagnostic performance. Multi-marker combinations were considered positive, if at least one marker in the combination was positive. A systematic search for the best n-marker combination (starting with n=2) was conducted in the following steps: i) calculate the Youden index, equaling the sum of sensitivity for detecting early stage CRC (UICC TNM stage I/II) and specificity minus 1 for all possible marker combinations; ii) determine the specific n-marker combination which gives the highest index and evaluate its diagnostic performance in the training set; iii) continue the selection process with increasing n, the number of markers included in the combination, until the specificity of the best performing marker combination in the training set falls below 90%. The diagnostic performance of the final marker-combination was then evaluated in the validation set. 95% confidence intervals (95% CIs) of sensitivity and specificity were calculated using the Wilson method [49].

All statistical analyses were performed with the statistical software R version 3.1.1 [50]. All tests were two-sided and p-values of 0.05 or less were considered to be statistically significant.

## SUPPLEMENTARY TABLES




