# Supplementary material for: Prospective evaluation of 64 serum autoantibodies as biomarkers for early detection of colorectal cancer in a true screening setting
Source: Oncotarget. 2016 Feb 19;7(13):16420–32. doi: 10.18632/oncotarget.7500 (PMC4941325; doi:10.18632/oncotarget.7500)
Supplement: Supplementary file 1 [file oncotarget-07-16420-s001.pdf]

SUPPLEMENTARY TABLES

Supplementary Table 1: Gene names and protein names of all 64 measured antigens

Synonyms	Protein full names	Protein length (amino acid sequence)
**ACRBP**	Acrosin-binding protein	full length
**AIMP1**	Aminoacyl tRNA synthase complex-interacting multifunctional protein 1	full length
**ANXA4**	Annexin A4	full length
**BIRC5**	Baculoviral IAP repeat-containing protein 5	full length
**CALU**	Calumenin	full length
**CAMEL**	CTL-recognized antigen on melanoma	full length
**CCNB1**	G2/mitotic-specific cyclin-B1	full length
**CCND1**	G1/S-specific cyclin-D1	full length
**CDKN2A**	Cyclin-dependent kinase inhibitor 2A	full length
**CEACAM5**	Carcinoembryonic antigen-related cell adhesion molecule 5	full length
**CT47A**	Cancer/testis antigen 47A	full length
**CTAG1**	Cancer/testis antigen 1	full length
**CTAG2**	Cancer/testis antigen 2	full length
**DDX53**	Probable ATP-dependent RNA helicase DDX53	full length
**EGFR_C_term**	Epidermal growth factor receptor	669–1210
**ERBB2_N_term**	Receptor tyrosine-protein kinase erbB-2	23–652
**ERBB2_C_term**	Receptor tyrosine-protein kinase erbB-2	676–1255
**FOLH1_iso1**	Glutamate carboxypeptidase 2 (isoform 1)	full length
**FOLH1_iso7**	Glutamate carboxypeptidase 2 (isoform 7)	full length
**GAGE7**	G antigen 7	full length
**GRINA**	Protein lifeguard 1	full length
**GRINA_N-term**	Protein lifeguard 1	1–164
**HIST1H2B**	Histone H2B type 1-C/E/F/G/I	full length
**HMGN3**	High mobility group nucleosome-binding domain-containing protein 3	full length
**HSPA2**	Heat shock-related 70 kDa protein 2	full length
**HSPA5**	78 kDa glucose-regulated protein	19-end
**IGF2BP1**	Insulin-like growth factor 2 mRNA-binding protein 1	full length
**IGF2BP3**	Insulin-like growth factor 2 mRNA-binding protein 3	full length
**IMPDH2**	Inosine-5′-monophosphate dehydrogenase 2	full length
**KLK3_iso1**	Prostate-specific antigen (isoform 1)	full length
**KLK3_iso2**	Prostate-specific antigen (isoform 2)	full length
**KRAS**	GTPase Kras	1–37
**MAGEA1**	Melanoma-associated antigen 1	full length

(*Continued*)

Synonyms	Protein full names	Protein length (amino acid sequence)
**MAGEA3**	Melanoma-associated antigen 3	full length
**MAGEA4**	Melanoma-associated antigen 4	full length
**MAPKAPK3**	MAP kinase-activated protein kinase 3	full length
**MDM2**	E3 ubiquitin-protein ligase Mdm2	full length
**MLANA**	Melanoma antigen recognized by T-cells 1	full length
**MPHOSPH6**	M-phase phosphoprotein 6	full length
**MIA**	Melanoma-derived growth regulatory protein	full length
**MTDH**	Protein LYRIC	271–451
**MUC1_iso8**	Mucin-1 (isoform 8)	full length
**MYC**	Myc proto-oncogene protein	full length
**PMEL**	Melanocyte protein PMEL	full length
**PSCA**	Prostate stem cell antigen	full length
**REG3A**	Regenerating islet-derived protein 3-alpha	27-end
**RPH3AL**	Rab effector Noc2	full length
**RPL13**	60S ribosomal protein L13	full length
**SNAP25**	Synaptosomal-associated protein 25	full length
**SAG**	S-arrestin	full length
**SDCCAG8**	Serologically defined colon cancer antigen 8	225–537
**SEC61B**	Protein transport protein Sec61 subunit beta	full length
**SPAG9**	C-Jun-amino-terminal kinase-interacting protein 4	full length
**SPANXA**	Sperm protein associated with the nucleus on the X chromosome A	full length
**SSX2**	Protein SSX2	full length
**SSX4**	Protein SSX4	full length
**TP53**	Cellular tumor antigen p53	full length
**TPM3_iso1**	Tropomyosin alpha-3 chain (isoform 1)	full length
**TPM3_iso3**	Tropomyosin alpha-3 chain (isoform 3)	full length
**TRP-2**	Tyrosinase-related protein-2	full length
**UBE2D1**	Ubiquitin-conjugating enzyme E2 D1	full length
**UBQLN1**	Ubiquilin-1	full length
**UCHL3**	Ubiquitin carboxyl-terminal hydrolase isozyme L3	full length
**VIL1**	Villin-1	full length

Supplementary Table S2: Diagnostic performance of all 64 single autoantibody markers for detecting colorectal neo-plasms

See Supplementary File 1
