# Supplementary material for: Prospective evaluation of 64 serum autoantibodies as biomarkers for early detection of colorectal cancer in a true screening setting
Source: Oncotarget. 2016 Feb 19;7(13):16420–32. doi: 10.18632/oncotarget.7500 (PMC4941325; doi:10.18632/oncotarget.7500)
Supplement: Supplementary file 2 [file oncotarget-07-16420-s002.odt]

| Supplementary Table 2. Diagnostic performance  of all 64 single autoantibody markers for detecting colorectal neoplasms |  |
| --- | --- |
| Antigen | cutoff (MFI) | Training set |  | Validation set |
| SE for CRC† | SE for stage I/II CRC† | SE for stage III/IV CRC† | SPE† |  | SE for CRC† | SE for stage 0/I/II CRC† | SE for stage III/IV CRC† | SE     for AA† | SE   for NA† |
| TP53 | 601 | 14 [11-18] | 13 [9-19] | 15 [10-21] | 98 [93-99] |  | 8 [3-19] | 7 [2-23] | 9 [3-28] | 1 [0-6] | 0 [0-12] |
| IMPDH2 | 47 | 8 [5-11] | 10 [7-15] | 5 [2-9] | 98 [93-99] |  | 4 [1-14] | 4 [0-18] | 5 [0-22] | 6 [3-13] | 7 [2-22] |
| MAGEA4 | 168 | 9 [6-12] | 9 [6-14] | 8 [5-14] | 98 [93-99] |  | 6 [2-17] | 11 [4-28] | 0 [0-15] | 12 [7-20] | 0 [0-12] |
| RPL13 | 170 | 8 [5-11] | 9 [5-13] | 6 [4-12] | 98 [93-99] |  | 4 [1-14] | 0 [0-12] | 9 [3-28] | 2 [1-7] | 10 [4-26] |
| MDM2 | 400 | 5 [3-8] | 8 [5-12] | 1 [0-5] | 98 [93-99] |  | 2 [0-11] | 4 [0-18] | 0 [0-15] | 1 [0-6] | 7 [2-22] |
| CTAG1 | 91 | 6 [4-9] | 7 [4-11] | 6 [3-11] | 98 [93-99] |  | 2 [0-11] | 0 [0-12] | 5 [0-22] | 6 [3-13] | 3 [0-17] |
| RPH3AL | 87 | 7 [5-10] | 7 [4-11] | 8 [5-13] | 98 [93-99] |  | 0 [0-7] | 0 [0-12] | 0 [0-15] | 6 [3-13] | 3 [0-17] |
| ANXA4 | 50 | 6 [4-9] | 6 [4-10] | 6 [4-12] | 98 [93-99] |  | 4 [1-14] | 4 [0-18] | 5 [0-22] | 2 [1-7] | 7 [2-22] |
| MTDH | 440 | 4 [2-6] | 6 [4-10] | 1 [0-4] | 98 [93-99] |  | 4 [1-14] | 4 [0-18] | 5 [0-22] | 2 [1-7] | 3 [0-17] |
| TPM3_iso1 | 424 | 7 [4-10] | 6 [4-10] | 7 [4-12] | 98 [93-99] |  | 8 [3-19] | 0 [0-12] | 18 [7-39] | 4 [2-10] | 7 [2-22] |
| ERBB2_C_term | 90 | 6 [4-9] | 6 [3-10] | 6 [4-12] | 98 [93-99] |  | 6 [2-17] | 7 [2-23] | 5 [0-22] | 8 [4-15] | 3 [0-17] |
| IGF2BP1 | 309 | 5 [3-8] | 6 [3-10] | 4 [2-8] | 98 [93-99] |  | 2 [0-11] | 4 [0-18] | 0 [0-15] | 2 [1-7] | 3 [0-17] |
| MIA | 52 | 4 [3-7] | 5 [3-9] | 3 [1-7] | 98 [94-100] |  | 2 [0-11] | 4 [0-18] | 0 [0-15] | 1 [0-6] | 3 [0-17] |
| DDX53 | 460 | 5 [3-8] | 5 [2-8] | 5 [3-10] | 98 [93-99] |  | 0 [0-7] | 0 [0-12] | 0 [0-15] | 2 [1-7] | 3 [0-17] |
| HMGN3 | 706 | 5 [3-8] | 5 [2-8] | 5 [3-10] | 98 [93-99] |  | 8 [3-19] | 11 [4-28] | 5 [0-22] | 9 [5-16] | 10 [4-26] |
| CALU | 578 | 4 [3-7] | 4 [2-8] | 5 [2-9] | 98 [93-99] |  | 6 [2-17] | 0 [0-12] | 14 [5-33] | 4 [2-10] | 3 [0-17] |
| KLK3_iso1 | 41 | 4 [2-6] | 4 [2-8] | 3 [1-7] | 98 [93-99] |  | 2 [0-11] | 4 [0-18] | 0 [0-15] | 1 [0-6] | 3 [0-17] |
| REG3A | 62 | 3 [2-6] | 4 [2-8] | 2 [1-6] | 98 [93-99] |  | 0 [0-7] | 0 [0-12] | 0 [0-15] | 1 [0-6] | 3 [0-17] |
| CTAG2 | 135 | 5 [3-7] | 4 [2-7] | 6 [3-11] | 98 [93-99] |  | 4 [1-14] | 7 [2-23] | 0 [0-15] | 3 [1-9] | 0 [0-12] |
| ERBB2_N_term | 81 | 3 [2-6] | 4 [2-7] | 3 [1-6] | 98 [93-99] |  | 4 [1-14] | 4 [0-18] | 5 [0-22] | 3 [1-9] | 3 [0-17] |
| MAGEA3 | 220 | 3 [2-6] | 4 [2-7] | 3 [1-7] | 98 [93-99] |  | 4 [1-14] | 7 [2-23] | 0 [0-15] | 6 [3-13] | 0 [0-12] |
| GRINA | 50 | 4 [2-7] | 3 [1-6] | 5 [3-10] | 98 [93-99] |  | 0 [0-7] | 0 [0-12] | 0 [0-15] | 2 [1-7] | 3 [0-17] |
| GRINA_N-term | 46 | 4 [2-7] | 3 [1-6] | 5 [3-10] | 98 [93-99] |  | 0 [0-7] | 0 [0-12] | 0 [0-15] | 3 [1-9] | 3 [0-17] |
| IGF2BP3 | 343 | 3 [1-5] | 3 [1-6] | 2 [1-6] | 98 [93-99] |  | 2 [0-11] | 0 [0-12] | 5 [0-22] | 2 [1-7] | 3 [0-17] |
| Continued on the following page |  |
| Supplementary Table 2. Continued |  |
| Antigen | cutoff (MFI) | Training set |  | Validation set |
| SE for CRC† | SE for stage I/II CRC† | SE for stage III/IV CRC† | SPE† |  | SE for CRC† | SE for stage 0/I/II CRC† | SE for stage III/IV CRC† | SE     for AA† | SE   for NA† |
| KRAS | 59 | 2 [1-4] | 3 [1-6] | 1 [0-5] | 98 [93-99] |  | 0 [0-7] | 0 [0-12] | 0 [0-15] | 2 [1-7] | 0 [0-12] |
| MAPKAPK3 | 286 | 3 [2-5] | 3 [1-6] | 3 [1-6] | 98 [93-99] |  | 2 [0-11] | 0 [0-12] | 5 [0-22] | 3 [1-9] | 0 [0-12] |
| SNAP25 | 1091 | 3 [2-5] | 3 [1-6] | 3 [1-6] | 98 [93-99] |  | 2 [0-11] | 0 [0-12] | 5 [0-22] | 1 [0-6] | 3 [0-17] |
| SAG | 828 | 2 [1-4] | 3 [1-6] | 1 [0-4] | 98 [93-99] |  | 2 [0-11] | 4 [0-18] | 0 [0-15] | 2 [1-7] | 0 [0-12] |
| UBQLN1 | 361 | 4 [2-6] | 3 [1-6] | 5 [2-9] | 98 [93-99] |  | 4 [1-14] | 7 [2-23] | 0 [0-15] | 0 [0-4] | 0 [0-12] |
| CT47A | 1155 | 1 [1-3] | 3 [1-6] | 0 [0-2] | 98 [93-99] |  | 2 [0-11] | 4 [0-18] | 0 [0-15] | 2 [1-7] | 0 [0-12] |
| EGFR_C_term | 114 | 2 [1-4] | 3 [1-6] | 1 [0-5] | 98 [93-99] |  | 2 [0-11] | 4 [0-18] | 0 [0-15] | 3 [1-9] | 3 [0-17] |
| FOLH1_iso1 | 35 | 2 [1-4] | 3 [1-6] | 1 [0-5] | 98 [93-99] |  | 0 [0-7] | 0 [0-12] | 0 [0-15] | 0 [0-4] | 0 [0-12] |
| KLK3_iso2 | 54 | 3 [2-5] | 3 [1-6] | 3 [1-7] | 98 [94-100] |  | 2 [0-11] | 4 [0-18] | 0 [0-15] | 0 [0-4] | 3 [0-17] |
| MAGEA1 | 478 | 2 [1-4] | 3 [1-6] | 1 [0-5] | 98 [93-99] |  | 2 [0-11] | 4 [0-18] | 0 [0-15] | 4 [2-10] | 0 [0-12] |
| MUC1_iso8 | 305 | 3 [2-6] | 3 [1-6] | 4 [2-8] | 98 [93-99] |  | 2 [0-11] | 4 [0-18] | 0 [0-15] | 2 [1-7] | 10 [4-26] |
| PSCA | 30 | 2 [1-4] | 3 [1-6] | 2 [1-6] | 100 [97-100] |  | 6 [2-17] | 0 [0-12] | 14 [5-33] | 0 [0-4] | 3 [0-17] |
| AIMP1 | 567 | 3 [1-5] | 2 [1-5] | 3 [1-7] | 98 [93-99] |  | 2 [0-11] | 4 [0-18] | 0 [0-15] | 2 [1-7] | 0 [0-12] |
| CCND1 | 126 | 1 [1-3] | 2 [1-5] | 1 [0-4] | 98 [93-99] |  | 2 [0-11] | 4 [0-18] | 0 [0-15] | 1 [0-6] | 0 [0-12] |
| CDKN2A | 30 | 1 [0-3] | 2 [1-5] | 0 [0-2] | 98 [94-100] |  | 0 [0-7] | 0 [0-12] | 0 [0-15] | 3 [1-9] | 0 [0-12] |
| FOLH1_iso7 | 30 | 1 [1-3] | 2 [1-5] | 1 [0-4] | 100 [97-100] |  | 0 [0-7] | 0 [0-12] | 0 [0-15] | 0 [0-4] | 0 [0-12] |
| HSPA2 | 687 | 3 [1-5] | 2 [1-5] | 3 [1-7] | 98 [93-99] |  | 6 [2-17] | 0 [0-12] | 14 [5-33] | 2 [1-7] | 3 [0-17] |
| MLANA | 618 | 1 [1-3] | 2 [1-5] | 1 [0-4] | 98 [93-99] |  | 0 [0-7] | 0 [0-12] | 0 [0-15] | 4 [2-10] | 0 [0-12] |
| MPHOSPH6 | 3206 | 1 [1-3] | 2 [1-5] | 1 [0-4] | 98 [93-99] |  | 2 [0-11] | 0 [0-12] | 5 [0-22] | 2 [1-7] | 3 [0-17] |
| MYC | 174 | 2 [1-4] | 2 [1-5] | 3 [1-6] | 98 [93-99] |  | 2 [0-11] | 4 [0-18] | 0 [0-15] | 4 [2-10] | 7 [2-22] |
| SDCCAG8 | 1023 | 3 [2-5] | 2 [1-5] | 4 [2-8] | 98 [93-99] |  | 2 [0-11] | 0 [0-12] | 5 [0-22] | 1 [0-6] | 7 [2-22] |
| SEC61B | 40 | 2 [1-4] | 2 [1-5] | 2 [1-6] | 98 [93-99] |  | 2 [0-11] | 0 [0-12] | 5 [0-22] | 1 [0-6] | 0 [0-12] |
| SPANXA | 5001 | 2 [1-4] | 2 [1-5] | 1 [0-5] | 98 [93-99] |  | 2 [0-11] | 4 [0-18] | 0 [0-15] | 0 [0-4] | 0 [0-12] |
| SSX4 | 69 | 4 [2-6] | 2 [1-5] | 6 [3-11] | 98 [93-99] |  | 2 [0-11] | 4 [0-18] | 0 [0-15] | 2 [1-7] | 7 [2-22] |
| Continued on the following page |  |
| Supplementary Table 2. Continued |  |
| Antigen | cutoff (MFI) | Training set |  | Validation set |
| SE for CRC† | SE for stage I/II CRC† | SE for stage III/IV CRC† | SPE† |  | SE for CRC† | SE for stage 0/I/II CRC† | SE for stage III/IV CRC† | SE     for AA† | SE   for NA† |
| TPM3_iso3 | 1036 | 2 [1-4] | 2 [1-5] | 3 [1-6] | 98 [93-99] |  | 2 [0-11] | 0 [0-12] | 5 [0-22] | 2 [1-7] | 7 [2-22] |
| UBE2D1 | 427 | 3 [1-5] | 2 [1-5] | 3 [1-7] | 98 [93-99] |  | 0 [0-7] | 0 [0-12] | 0 [0-15] | 0 [0-4] | 0 [0-12] |
| CCNB1 | 2106 | 1 [0-3] | 2 [1-4] | 1 [0-4] | 98 [93-99] |  | 2 [0-11] | 0 [0-12] | 5 [0-22] | 1 [0-6] | 0 [0-12] |
| GAGE7 | 1665 | 2 [1-4] | 2 [1-4] | 3 [1-6] | 98 [93-99] |  | 0 [0-7] | 0 [0-12] | 0 [0-15] | 1 [0-6] | 0 [0-12] |
| HSPA5 | 43 | 1 [1-3] | 2 [1-4] | 1 [0-5] | 98 [93-99] |  | 2 [0-11] | 4 [0-18] | 0 [0-15] | 1 [0-6] | 3 [0-17] |
| ACRBP | 3582 | 1 [0-2] | 1 [0-4] | 0 [0-2] | 98 [93-99] |  | 0 [0-7] | 0 [0-12] | 0 [0-15] | 0 [0-4] | 0 [0-12] |
| BIRC5 | 1283 | 1 [0-2] | 1 [0-4] | 1 [0-4] | 98 [93-99] |  | 0 [0-7] | 0 [0-12] | 0 [0-15] | 1 [0-6] | 0 [0-12] |
| CAMEL | 133 | 1 [0-3] | 1 [0-4] | 1 [0-5] | 98 [93-99] |  | 2 [0-11] | 4 [0-18] | 0 [0-15] | 0 [0-4] | 0 [0-12] |
| PMEL | 78 | 2 [1-4] | 1 [0-4] | 4 [2-8] | 98 [93-99] |  | 4 [1-14] | 7 [2-23] | 0 [0-15] | 4 [2-10] | 0 [0-12] |
| SPAG9 | 693 | 2 [1-4] | 1 [0-4] | 4 [2-8] | 98 [93-99] |  | 4 [1-14] | 7 [2-23] | 0 [0-15] | 10 [6-18] | 3 [0-17] |
| TRP-2 | 30 | 1 [0-2] | 1 [0-4] | 0 [0-2] | 100 [97-100] |  | 0 [0-7] | 0 [0-12] | 0 [0-15] | 0 [0-4] | 0 [0-12] |
| UCHL3 | 645 | 1 [0-2] | 1 [0-4] | 0 [0-2] | 98 [93-99] |  | 0 [0-7] | 0 [0-12] | 0 [0-15] | 0 [0-4] | 3 [0-17] |
| HIST1H2B | 2234 | 1 [0-2] | 1 [0-3] | 1 [0-4] | 98 [93-99] |  | 0 [0-7] | 0 [0-12] | 0 [0-15] | 0 [0-4] | 0 [0-12] |
| SSX2 | 774 | 1 [0-2] | 1 [0-3] | 1 [0-5] | 98 [93-99] |  | 2 [0-11] | 4 [0-18] | 0 [0-15] | 0 [0-4] | 0 [0-12] |
| VIL1 | 1909 | 1 [0-2] | 1 [0-3] | 1 [0-4] | 98 [93-99] |  | 2 [0-11] | 4 [0-18] | 0 [0-15] | 4 [2-10] | 7 [2-22] |
| CEACAM5 | 316 | 0 [0-2] | 0 [0-2] | 1 [0-4] | 98 [93-99] |  | 0 [0-7] | 0 [0-12] | 0 [0-15] | 2 [1-7] | 0 [0-12] |

† In % with 95% confidence interval.

Abbreviations: AA, advanced adenoma; NA, non−advanced adenoma; CRC, colorectal cancer, SE, sensitivity; SPE, specificity.
